# Predictive model for postoperative pleural effusion after hepatectomy

**DOI:** 10.1002/ags3.12417

**Published:** 2020-12-17

**Authors:** Hidetoshi Nitta, Chisho Mitsuura, Yuta Shiraishi, Tatsunori Miyata, Kenji Shimizu, Kazuto Harada, Ryuichi Karashima, Toshiro Masuda, Katsutaka Matsumoto, Tetsuya Okino, Yo‐ichi Yamashita, Hideo baba, Hiroshi Takamori

**Affiliations:** ^1^ Department of Surgery Saiseikai Kumamoto Hospital Kumamoto Japan; ^2^ Department of Gastroenterological Surgery Graduate School of Medical Sciences Kumamoto University Kumamoto Japan

**Keywords:** hepatectomy, pleural effusion, preventive thoracic drainage

## Abstract

**Aim:**

Severe postoperative pleural effusion (sPOPE) after hepatectomy can lead to respiratory distress and may require thoracic drainage, leading to prolonged hospitalization. Preventive chest tube insertion may be useful for patients at high risk for sPOPE. We aimed to develop a predictive model for sPOPE after hepatectomy and evaluate indications for preventive chest tube insertion using our model.

**Methods:**

We evaluated all patients who underwent hepatectomy from 2013 to 2020. Risk factors for sPOPE were used to develop a predictive model for sPOPE, which was validated in a cohort that received preventative chest tube placement postoperatively.

**Results:**

A total of 325 patients were analyzed. Thirty‐one (9.5%) patients had a preventive chest tube placed at the end of their operation. Twenty‐one patients out of the remaining 294 patients developed sPOPE. Multivariate analysis identified resection containing segment 8 [relative risk (RR) 3.24, *P* = .022], intraoperative bleeding ≥ 500 g (RR 4.02, *P* = .008), intraoperative diaphragmatic incision (RR 6.96, *P* = .042) and open hepatectomy (RR 7.51, *P* = .016) as independently associated with sPOPE. The estimated probability of sPOPE ranged from 0.4% in patients with none of these factors to 73.4% in the presence of all factors. Among the 31 patients who received a preventive chest tube, more patients in the high‐risk group defined by the model had postoperative pleural effusions compared to the low‐risk group (*P* = .012).

**Conclusion:**

Our predictive model for sPOPE using four risk factors allows for reliable prediction and may be useful for selection of preventive chest tube in patients undergoing hepatectomy.

## Graphical abstract

We established a predictive model for severe posthepatectomy pleural effusion (sPOPE). Our predictive model of sPOPE using four risk factors allows for reliable prediction and may be useful for selection of preventive chest tube in patients undergoing hepatectomy.

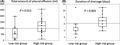

## 1 INTRODUCTION

Although hepatectomy has become a relatively safe operative procedure, the emergence of pleural effusion afterwards is one of the commonly observed complications, occurring in 18%‐71% of cases.^1^, ^2^, ^3^, ^4^ ^.^ The clinical presentation of postoperative pleural effusion (POPE) is wide and ranges from asymptomatic to respiratory distress with dyspnea or respiratory failure requiring reintubation and mechanical ventilation.^5^, ^6^, ^7^ There are several studies pertaining to the risk factors of posthepatectomy pleural effusion.^2^, ^8^, ^9^, ^10^ However, most of these studies analyzed not only severe pleural effusion but also mild pleural effusion managed with noninvasive therapy, such as fluid or salt restriction, and diuretic agent administration. Controllable, or mild, effusion is not so problematic in clinical practice. However, symptomatic pleural effusion requiring thoracic drainage accounts for 9.4% of cases.^1^ Such severe POPE (sPOPE) influences patient outcomes by delaying postoperative recovery and leading to increased hospital stay and higher associated costs.^2^, ^10^ Therefore, analysis of risk factors for sPOPE is of strategic significance for postoperative management. However, there are currently little data available regarding risk factors of sPOPE and its management, making management and prevention a challenge for clinicians.

Intraoperative chest tube insertion and continuous drainage of the right thoracic cavity may be effective to prevent sPOPE for high‐risk patients as nearly all sPOPE post‐hepatectomy develops within the right thoracic cavity.^8^ Prevention of pleural effusions post‐hepatectomy may allow for improved pulmonary function and less subjective patient dyspnea, thus promoting postoperative rehabilitation and better postoperative course. However, no reports or guidelines currently exist on the role of preventative chest tube placement for hepatectomy patients or which patients may benefit from this intervention. Our study, therefore, aims to identify independent predictors of sPOPE and establish a model to predict sPOPE based on pre‐ or intraoperative valuables. We then aim to evaluate candidates for preventive chest tube placement using our model.

## 2 METHODS

### 2.1 Patient population

A total of 325 consecutive patients who underwent hepatectomy at Saiseikai Kumamoto Hospital between January 2013 and April 2020 were enrolled in this study. Patients who underwent simultaneous procedures (n = 47) such as cholecystectomy, colectomy, biliary reconstruction for biliary cancer, or gastrectomy were also included. This study was approved by the Institutional Review Board at Saiseikai Kumamoto Hospital. Patients who underwent hepatectomy without preventive chest tube insertion at Kumamoto University between June 2012 and June 2015 were assigned to a validation cohort (n = 290).

### 2.2 Surgical procedure

Of the patients, 204 underwent laparotomies and 121 underwent laparoscopic hepatic resections. Tumor location, size, and its relationship to the portal and hepatic veins were examined using intraoperative ultrasound, regardless of whether laparotomy or laparoscopic methods were used. Hepatic parenchymal transection was performed with a Cavitron Ultrasonic Surgical Aspirator (Olympus, Tokyo, Japan) or clamp crush technique. Twenty‐two patients required diaphragmatic incision intraoperatively due to large right‐sided tumors, tumor invasion to the diaphragm, or adhesiolysis between the diaphragm and liver. 31 patients (9.5%) had a chest tube (20 Fr thoracic tube) inserted at the 9th or 10th intercostal space in the anterior axillary or mid‐axillary line in the right thoracic cavity at the end of the operation for prevention of sPOPE. The chest tube was connected to a MERA continuous suction unit (Senko Medical Instrument, Tokyo, Japan) with drainage under negative pressure from the immediate postoperative period. The indication for preventive chest tube insertion was decided by each surgeon’s personal assessment of the risk for sPOPE.

Resections containing segment 8 (S8) area included partial resection, S8 segmentectomy, anterior sectionectomy, central bisectionectomy and right hemihepatectomy.

### 2.3 Pleural effusion

Postoperative pleural effusion was evaluated by chest X‐ray on postoperative days 1 and 3, or computed tomography (CT) at postoperative day 5, for all patients. Once pleural effusion was detected by imaging, clinical evaluation including pulmonary auscultation and oxygen saturation monitoring were carried out. Patients showing signs of dyspnea or respiratory distress received diuretics. sPOPE was defined as accumulation of moderate to large pleural effusion with symptoms regardless of diuretic agent administration and was managed with ultrasound‐guided chest tube insertion with continuous drainage using a MERA continuous suction unit (Senko Medical Instrument). Daily chest tube output was monitored and the chest tube was removed when output was less than 100 mL/day. The same criteria for chest tube removal applied to patients who underwent preventive chest tube insertion at the end of their operation.

### 2.4 Statistical analysis

Categorical variables were compared using χ^2^ or Fisher’s exact tests. Continuous variables are depicted as mean ± standard deviation (SD). Continuous variables were compared using Mann–Whitney *U* tests. Variables that showed a *P*‐value of <0.05 on univariate analysis were included in our multivariate analysis using a logistic regression model. All variables associated with sPOPE were candidates using a stepwise backward elimination procedure with a threshold of *P < *.05. The level of significance for all tests was set at *P* < .05. The predictive performance of the model was measured by the area under the curve of the receiver operating characteristic (ROC) curve analysis. The percentages of sPOPE probability in the predictive model were calculated based on the coefficients that were obtained by the multivariate logistic regression model. All statistical analyses were carried out using JMP version 12 (SAS Institute, Cary, NC, USA), and R version 3.1.1.

## 3 RESULTS

### 3.1 Patient characteristics

Baseline characteristics of all included patients (n = 325) are shown in Table 1. One of 31 patients with preventive chest tube developed sPOPE after removal of the chest tube and required chest tube reinsertion. Among the 294 patients without preventive chest tube, 21 patients developed sPOPE requiring thoracic drainage at median postoperative day 6 (1‐52). Twenty patients out of the 21 patients underwent drainage of the right thoracic cavity and one patient in the left thoracic cavity. No patients developed complications associated with chest tube insertion. Mean postoperative hospital stay of patients without sPOPE (n = 273), with sPOPE (n = 21), and with preventive chest tube were 10 ± 7, 29 ± 31 and 13 ± 9 days, respectively. Postoperative hospital stay of patients with sPOPE was significantly longer than that of patients without sPOPE (*P* < .0001) and patients with preventive chest tube (*P* = .002). Baseline characteristics of the validation cohort (n = 290) are shown in Table S1.

**Table 1:** Clinical characteristics of patients in the current cohort

	Without preventive chest tube n = 294	Preventive chest tube n = 31
Male : Female	230:64	20:11
Age, y (±SD)	69 (±11)	69 (±10)
Body mass index (±SD)	23.0 (±3.8)	24.5 (±3.5)
Primary disease		
HCC : CRLM : Biliary cancer : Other	159:75:39:21	15:7:5:4
HBsAg	35 (12%)	3 (10%)
HCV‐Ab	54 (18%)	7 (23%)
ALBI grade 1:2:3	212:82:0	17:14:0
Type of hepatectomy		
Partial	162 (55%)	9 (29%)
1 segment	31 (11%)	6 (19%)
2 segments	53 (18%)	1 (3%)
3 segments	29 (10%)	5 (16%)
≥4 segments	19 (6%)	10 (32%)
Open : Laparoscopic	174:120	29:2
Intraoperative diaphragm incision	7 (2%)	14 (45%)
Simultaneous procedure	47 (16%)	5 (16%)
Biliary reconstruction	22 (7%)	3 (10%)
Colectomy	16 (5%)	1 (3%)
Other	9 (3%)	1 (3%)
Complications (Clavien‐Dindo ≥ 3)	50 (17%)	9 (29%)
sPOPE	21 (7%)	1 (3%)
Bile leakage	22 (7%)	6 (19%)
Other	13 (4%)	2 (6%)
Operative death	2 (1%)	0 (0%)
Postoperative hospital stay (days)	10 ± 7	13 ± 9

Abbreviations: ALBI, albumin‐bilirubin; HBsAg, hepatitis B surface antigen; HCC, hepatocellular carcinoma; HCV‐Ab, hepatitis C virus antibody; sPOPE, severe postoperative pleural effusion.

### 3.2 Risk factors of sPOPE

We initially evaluated risk factors for sPOPE among patients without preventive chest tube. Univariate analysis revealed that serum albumin, tumor size > 30mm, hepatectomy of 1 segment or more, 2 segments or more, resection containing S8 area, open hepatectomy, intrahepatic diaphragm incision, operation time ≥ 360 min, and intraoperative blood loss ≥ 500mL were associated with sPOPE (Table 2). Multivariate analysis identified resection containing S8 area [relative risk (RR), 3.24; 95% confidence interval (CI),1.18‐9.58; *P = *.002], intraoperative blood loss ≥ 500 mL (RR, 4.02; 95%CI, 1.42‐12.56; *P* = .008), intraoperative diaphragm incision (RR, 6.96; 95%CI, 1.08‐44.70; *P* = .042), and open hepatectomy (RR, 7.51; 95%CI, 1.37‐139.95; *P* = .016) were independent risk factors for sPOPE (Table 3).

**Table 2:** Univariate analysis of risk factors for sPOPE

		Non‐sPOPE n = 273	sPOPE n = 21	*P*‐value
Age (y)	>70	155 (56.8%)	13 (61.9%)	.646
Gender	Male : Female	214:59	16:5	.816
Body mass index	≥25	92 (33.7%)	8 (38.1%)	.685
HBsAg		34 (12.5%)	1 (4.8%)	.243
HCV‐Ab		51 (18.7%)	3 (14.3%)	.606
Diabetes		87 (31.9%)	8 (38.1%)	.562
HCC : Non‐HCC		147:126	12:9	.770
%VC	<80%	23 (8.4%)	3 (14.3%)	.396
FEV1.0	<70%	49 (18.0%)	2 (9.5%)	.293
ASA‐PS	≥3	53 (19.4%)	4 (20.0%)	.994
BUN	≥20 mg/dL	43 (15.8%)	5 (23.8%)	.359
Creatinine	≥1.0 mg/dL	47 (17.2%)	2 (9.5%)	.331
Platelet count	<17 × 10^3^mm	96 (35.2%)	11 (52.4%)	.225
Serum albumin	<3.5 g/dL	26 (9.5%)	0 (0%)	.044
Total bilirubin	>1.0 mg/dL	54 (19.8%)	5 (23.8)	.663
Prothrombin time	<70%	23 (8.4%)	3 (14.3%)	.396
ICG R15	>10%	135 (49.4%)	13 (61.9%)	.269
Child‐Pugh	B	13 (4.8%)	2 (9.5%)	.386
ALBI grade	≥2	74 (27.1%)	8 (38.1%)	.293
Tumor size	>30 mm	95 (34.8%)	14 (66.7%)	.004
1 segment or more		117 (42.9%)	15 (71.4%)	.011
2 segments or more		89 (32.6%)	12 (57.1%)	.027
3 segments or more		45 (16.5%)	4 (19.1%)	.765
Resection containing S8		79 (28.9%)	14 (66.7%)	<.001
Resection containing S7		33 (12.1%)	6 (28.6%)	.054
Right lobectomy		15 (5.5%)	3 (14.3%)	.157
Left lobectomy		27 (9.9%)	0 (0%)	.040
Open : Laparoscopy		154:119	20:1	<.001
Diaphragm incision		4 (1.5%)	3 (14.3%)	.007
Simultaneous procedure		42 (15.4%)	5 (23.8%)	.335
Operation time	>360 min	67 (24.5%)	12 (57.1%)	.002
Intraoperative blood loss	>500 mL	62 (22.7%)	15 (71.4%)	<.001
Transfusion		23 (8.4%)	4 (19.1%)	.145
Bile leakage		18 (6.6%)	3 (14.3%)	.265
Makuuchi criteria	Out	42 (15.4%)	6 (28.6%)	.143

Abbreviations: ALBI, albumin‐bilirubin; ASA‐PS, American Society of Anesthesiologists physical status; BUN, blood urea nitrogen; FEV1.0, forced expiratory volume at 1 second on spirogram; HBsAg, hepatitis B surface antigen; HCC, hepatocellular carcinoma; HCV‐Ab, hepatitis C virus antibody; IC R15, indocyanine green retention rate at 15 min; sPOPE, severe postoperative pleural effusion; %VC, % vital capacity on spirogram.

**Table 3:** Multivariate analysis of risk factors for sPOPE

	Relative risk	95%CI	*P*‐value
Resection containing S8	3.24	1.18‐9.58	.022
Intraoperative blood loss ≥ 500 g	4.02	1.42‐12.56	.008
Intraoperative diaphragm incision	6.96	1.08‐44.70	.042
Open	7.51	1.37‐139.95	.016

Abbreviation: sPOPE, severe postoperative pleural effusion.

### 3.3 Predictive model for sPOPE

Per the predictive model based on the results of our logistic regression analysis, the probability of developing sPOPE was calculated using the following formula:

Score = 2.245 + 0.588[1(not S8 containing) or −1(S8 containing)] + 0.971[1(no diaphragm incision) or −1(diaphragm incision)]+1.008[1(laparoscopic) or −1(open)]+0.697[1(blood loss < 500 g) or −1 (blood loss ≥ 500 g)].

Probability (%) = 100 / [1 + exp(Score)].

**Figure 1:**
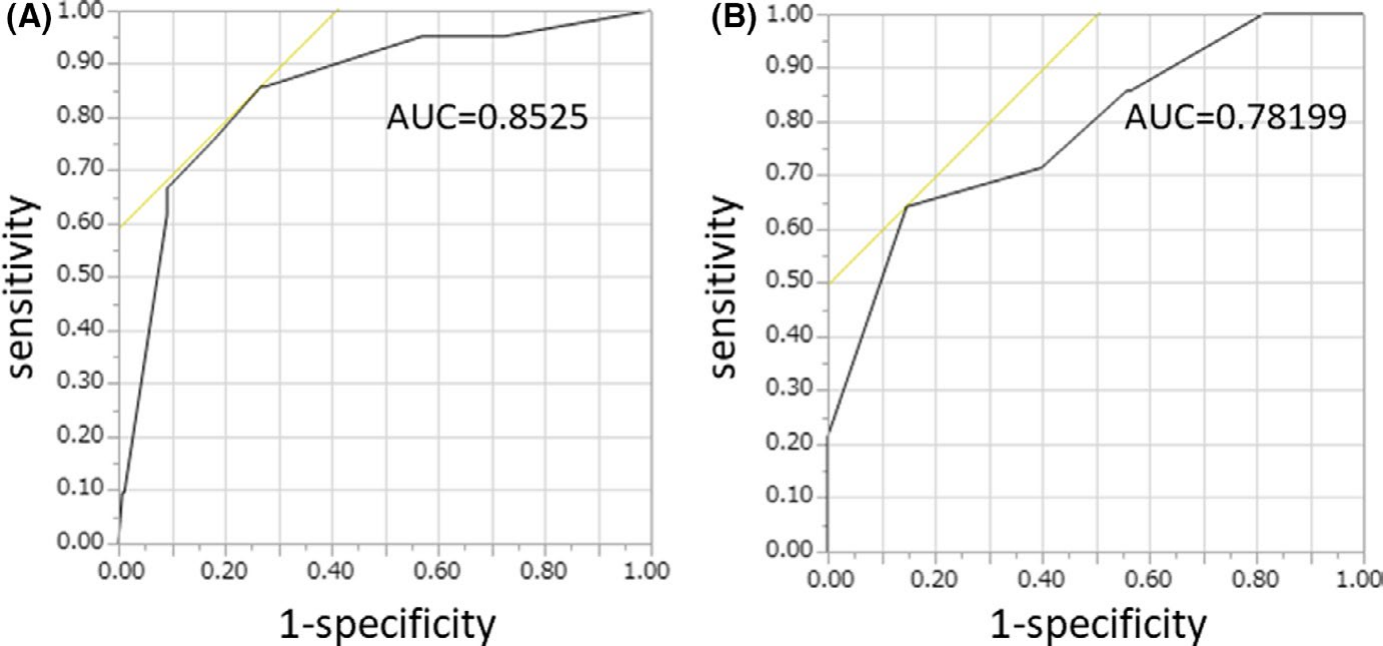
Receiver operating characteristic (ROC) curve analysis of the predictive model for severe postoperative pleural effusion (sPOPE). A, ROC curve of the current cohort. B, ROC curve of the validation cohort

For patients not presenting with any factors, the probability of sPOPE was 0.4%. The addition of each subsequent factor increased the risk to 2.9%, 17.4%, 46.0%, and 73.4% (Table 4). The c‐index, a measure of model discrimination represented by the area under the ROC curve, was 0.853 (Figure 1A). The model was then validated using the validation cohort. The c‐index was 0.782 (Figure 1B).

**Table 4:** Predictive model for sPOPE based on multivariate logistic regression analysis

	Open	Diaphragm incision	Blood loss ≥ 500 g	Resection containing S8	Probability (%)
4	+	+	+	+	73.4
3	+	+	+	−	46.0
	+	+	−	+	40.7
	+	−	+	+	28.4
	−	+	+	+	26.9
2	+	+	−	−	17.4
	+	−	+	−	10.9
	+	−	−	+	9.0
	−	+	+	−	10.2
	−	+	−	+	8.4
	−	−	+	+	5.0
1	+	−	−	−	2.9
	−	+	−	−	2.7
	−	−	+	−	1.6
	−	−	−	+	1.3
0	−	−	−	−	0.4
					

Abbreviation: sPOPE, severe postoperative pleural effusion.

### 3.4 Risk classification of POPE using the predictive model

**Figure 2:**
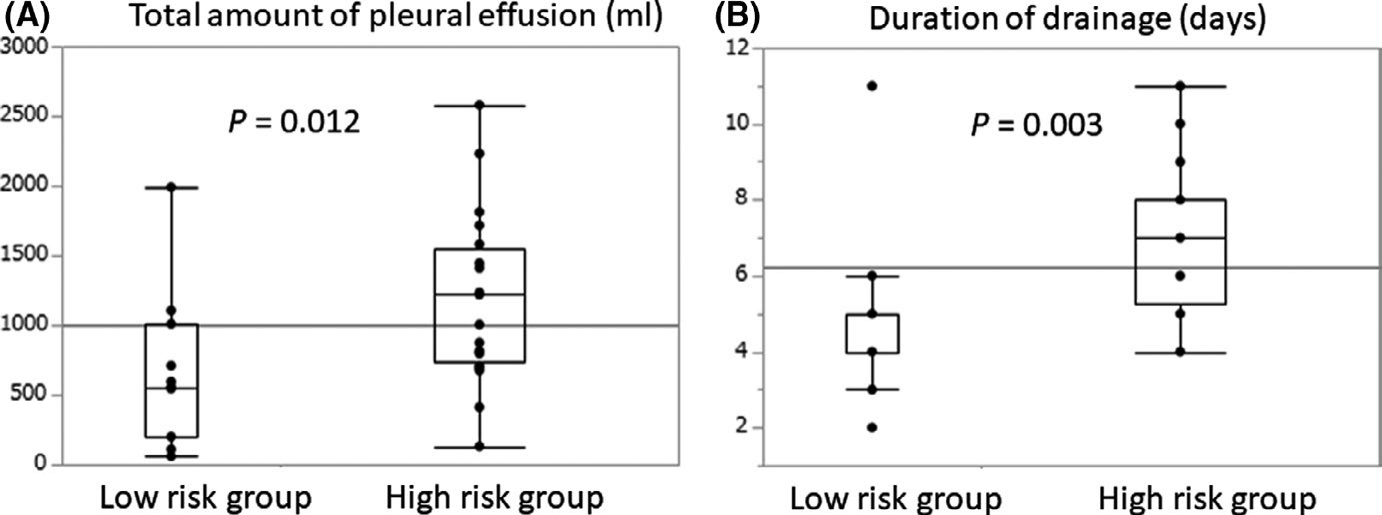
Comparison of total amount of drained pleural effusion (mL) and duration of preventive chest tube placement (days) between low‐ and high‐risk groups. A, Total amount of drained pleural effusion (mL) in the high‐risk group was significantly greater than that of the low‐risk group (*P* = .012). B, Duration (days) of preventive chest tube insertion in the high‐risk group was longer than that of the low‐risk group (*P* = .003)

**Figure 3:**
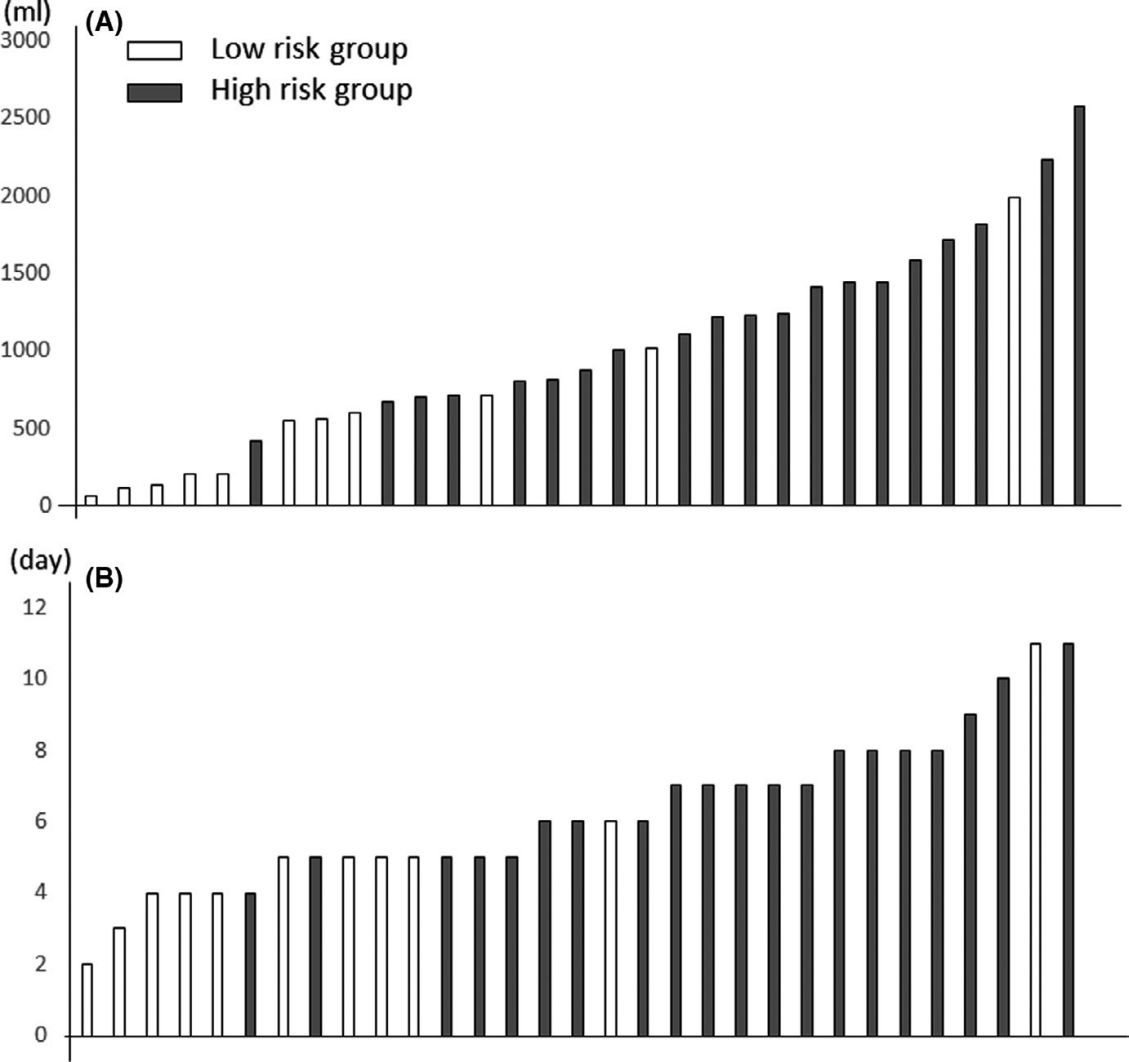
A, Total amount of drained (mL) pleural effusion in patients who underwent preventive chest tube insertion (n = 31) according to risk classification. B, Duration (days) of drainage for pleural effusion for patients who underwent preventive chest tube insertion (n = 31) according to risk classification

The optimal cut‐off value of the sPOPE probability was 26.9% by ROC analysis (sensitivity, 0.62; specificity 0.91). We therefore compared the short‐term outcomes of the 31 patients who underwent preventive chest tube insertion with sPOPE probabilities < 26.9% (low‐risk group, n = 11) and ≥ 26.9% (high‐risk group, n = 20). Total amount of drained pleural effusion until tube removal was higher in the high‐risk group (1200 ± 602 mL) than in the low‐risk group (643 ± 565mL; *P* = .012) (Figure 2A). Duration of chest tube insertion was longer in the high‐risk group (7.0 ± 1.8 days) compared to the low‐risk group (4.9 ± 2.3 days, *P* = .003) (Figure 2B). Moreover, 6 patients (55%) out of the 11 patients classified as low risk drained less than 500 mL of pleural fluid (Figure 3A). Five patients (45%) out of the 11 low‐risk patients had their chest tube removed within 4 days of placement (Figure 3B). In contrast, 18 patients (90%) out of 20 patients classified as high risk drained over 500ml of pleural fluid. Nineteen patients (95%) out of the 20 high‐risk patients required drainage for more than 5 days.

## 4 DISCUSSION

Postoperative pleural effusion after hepatectomy is a common postoperative complication and occurs in 18% to 71% of patients.^1^, ^2^, ^3^, ^4^ Symptomatic pleural effusion requiring thoracic drainage accounts for 9.4% of cases.^1^ In our study, 21 patients (7.1%) who developed sPOPE required drainage with prolonged hospital stay. We identified four independent risk factors for sPOPE: open hepatectomy, resection containing S8 area, blood loss more than 500 mL and intraoperative diaphragmatic incision. Combination of these four factors allows for reliable prediction of sPOPE in both the current cohort (c‐index, 0.853) and in the validation cohort (c‐index, 0.782).

All the risk factors were intraoperative variables, such as resected location, blood loss amount, operative methods, and diaphragmatic incision. There was no report on analysis for the resected segment per the Couinaud classification. Liver resection containing the S8 area was one of the risk factors of sPOPE in our study. Although the precise mechanism of POPE is still unknown, possible causes include irritation of the diaphragm due to rotation of right hepatic lobes or adhesiolysis between the diaphragm and liver, disruption of diaphragmatic lymphatics during liver mobilization involving sections of peritoneal diaphragmatic ligaments, or diaphragmatic defect by incision of diaphragm which allows the transfer of ascites directly into the pleural space.^11^, ^12^ The S8 area is widely in contact with the diaphragm. Resection of S8 requires extensive attention to the diaphragm, often involving incision of the diaphragm due to tumor size, invasion of tumor into the diaphragm, or the presence of adhesions all of which can lead to diaphragmatic irritation and increased risk of sPOPE.

Significant POPE after hepatectomy can lead to respiratory distress due to atelectasis and increased risk of pulmonary complications, such as pneumonia.^2^ Moreover, pulmonary complications are associated with increased perioperative death and other complications, as well as delayed postoperative rehabilitation, ultimately prolonging hospital stay^2^, ^13^ ^.^ Therefore, prediction and prevention of sPOPE is meaningful for patients’ postoperative course. However, preventive tube insertion itself carries risks of bleeding and lung injury and may not be appropriate for all patients. Therefore, definitive criteria of preventive tube insertion after hepatectomy are necessary. In our study, the median postoperative hospital stay of patients with sPOPE was significantly longer than that of patients who received a preventative chest tube. One of the reasons for the prolonged hospital stay is due to delayed start of pleural effusion drainage at median postoperative day 6. Therefore, preventing pleural effusion by chest tube insertion intraoperatively may be effective for improving overall postoperative course and shortening postoperative hospital stay. Based on the results of this study, patients having a probability of 26.9% or more may be good candidates for preventive tube insertion. Based on the results of our study, we aim to further validate these outcomes using prospective analysis of preventative chest tube placement in high‐risk patients undergoing hepatectomy.

We acknowledge the limitations of this study. First, it was a retrospective, single‐center study. Our results revealed a shorter postoperative stay for sPOPE patients who received a preventative chest tube, showing benefit of preventative chest tube insertion in patients with three or more risk factors. However, efficacy of preventative chest tube placement requires a prospective randomized controlled trial for further evaluation. Second, the condition of continuous drainage using a MERA continuous suction unit (Senko Medical Instrument) varied according to the attending physician, which may have some effect on the amount of drained pleural effusion. Third, the data which were used to establish the predictive model did not involve patients with preventive chest tube insertion, and it could be argued this allowed for some statistical bias.

## 5 CONCLUSION

Severe postoperative pleural effusion causes prolongation of hospital stay. Our predictive model consisting of four risk factors allows for reliable prediction of sPOPE. This model may be a valuable tool in deciding when to place a preventive chest tube in patients undergoing hepatectomy.

## CONFLICTS OF INTEREST

The authors declare no conflicts of interest.

## Supporting information

Supplementary Material

Supplementary Material
