# Supplementary material for: Predictive model for postoperative pleural effusion after hepatectomy
Source: Ann Gastroenterol Surg. 2020 Dec 17;5(3):373–80. doi: 10.1002/ags3.12417 (PMC8164455; doi:10.1002/ags3.12417)
Supplement: Supplementary file 1 — Supplementary Material [file AGS3-5-373-s002.docx]

**Supporting Information**

Supplementary Table

Baseline characteristics of patients in the validation cohort

|  | **Validation cohort n=290** |
| --- | --- |
| Male : female | 200:90 |
| Age (±SD) | 66 (±11) |
| Body mass index (±SD) | 23.0 (±3.5) |
| Primary disease  HCC：CRLM：biliary cancer：others | 189:42:20:39 |
| HBs-Ag  HCV-Ab | 46 (16%)  87 (30%) |
| ALBI grade 1:2:3 | 173:117:0 |
| Type of hepatectomy  Partial  1 segment  2 segments  3 segments  ≥4 segments | 138 (48%)  42 (14%)  67 (23%)  20 (7%)  23 (8%) |
| Open : laparoscopic | 209:81 |
| Intraoperative diaphragm incision | 5 (2%) |
| Simultaneous procedure  biliary reconstruction  colectomy  others | 14 (5%)  4 (1%)  6 (2%)  4 (1%) |
| Complications (Clavien dindo ≥3)  sPOPE  Bile leakage  others  operative death | 40 (14%)  13 (4%)  23 (8%)  4 (1%)  3 (1%) |
| Postoperative hospital stay (days) | 16±14 |

Abbreviations: HCC, hepatocellular carcinoma; HBs-Ag, hepatitis B surface antigen; HCV-ab, hepatitis C virus antibody; ALBI, albumin-bilirubin; sPOPE, severe postoperative pleural effusion
