# Supplementary material for: Predictive model for postoperative pleural effusion after hepatectomy
Source: Ann Gastroenterol Surg. 2020 Dec 17;5(3):373–80. doi: 10.1002/ags3.12417 (PMC8164455; doi:10.1002/ags3.12417)
Supplement: Supplementary file 2 — Supplementary Material [file AGS3-5-373-s001.docx]

**Supplementary Table Legend**

Baseline characteristics of patients in the validation cohort
